# The estimation of probability distribution for factor variables with many categorical values

**DOI:** 10.1371/journal.pone.0202547

**Published:** 2018-08-24

**Authors:** Minhyeok Lee, Yeong Seon Kang, Junhee Seok

**Affiliations:** 1 School of Electrical Engineering, Korea University, Seongbuk-gu, Seoul, South Korea; 2 Department of Business Administration, University of Seoul, Dongdaemun-gu, Seoul, South Korea; University of Nevada Reno, UNITED STATES

## Abstract

With recent developments of data technology in biomedicine, factor data such as diagnosis codes and genomic features, which can have tens to hundreds of discrete and unorderable categorical values, have emerged. While considered as a fundamental problem in statistical analyses, the estimation of probability distribution for such factor variables has not studied much because the previous studies have mainly focused on continuous variables and discrete factor variables with a few categories such as sex and race. In this work, we propose a nonparametric Bayesian procedure to estimate the probability distribution of factors with many categories. The proposed method was demonstrated through simulation studies under various conditions and showed significant improvements on the estimation errors from the previous conventional methods. In addition, the method was applied to the analysis of diagnosis data of intensive care unit patients, and generated interesting medical hypotheses. The overall results indicate that the proposed method will be useful in the analysis of biomedical factor data.

## Introduction

Factor variables are a common data type in statistical analysis of biomedical data. Distinct from continuous variables that can have infinite numbers of orderable values, a factor variable is characterized by a finite set of values or categories that are not orderable. Factor variables that have been considered in traditional biomedical data analyses, such as sex, race, and treatment options, usually have only a few categories. The number of categorical values is often much smaller than the size of observed samples.

Recently, with technology developments of data generation and accumulation, factor variables that can have many categorical values have emerged in the analyses of various biomedical data. For example, a diagnosis for a patient in electronic health records is represented as a factor variable having one of the thousands of diagnosis codes. The International Classification of Diseases version 10 (ICD-10), which is widely used for the standard diagnostic tool for health management, provides 155,000 different diagnosis codes [1]. Electronic health records of many clinical sites also include medical operations and prescribed drugs that can be similarly described by factor variables with thousands of categorical values. Molecular and genomic data are another example. A protein is a sequence of 20 distinct amino acids. The analysis of *k*-tuples of protein sequence motifs [2, 3] needs to be performed over 20^*k*^ distinct combinations of amino acids. In the high-throughput mRNA sequencing (RNA-Seq) data analysis [4, 5], each piece of RNA molecules is assigned to one of genomic regions. For human samples with about 20,000 genes, mRNA-Seq data can be represented as factor variables with 20,000 distinct values. In the natural language processing of biomedical literatures [6], a word is modeled as a factor variable with million choices.

Estimating the joint probability distribution of variables from observed random samples is a fundamental task in data analysis. For traditional factor variables with a few categories, the probability distribution can be efficiently estimated by maximum likelihood estimation (MLE), which can be calculated by counting samples falling into each combination of categories, because the sample size is assumed to be large enough compared to the number of possible combinations in most cases. However, this task is very challenging for the newly emerging factor variables with many categorical values because of the relative sparseness of observed samples. For example, the combination of two medical diagnosis encoded by ICD-10 has 24 billion choices, which are more than three times of the whole human population.

For the estimation of the probability distribution from observed samples, kernel estimation techniques have been extensively studied [7–13]. These techniques commonly employ kernel functions that smooth probability densities by borrowing supports from the adjacent data points to overcome the sparseness of samples. Since observed values should be orderable to measure the adjacency between data points, kernel techniques are usually applicable to only continuous variables [7–9] and ordinal factor variables [10, 11]. While some previous works have proposed kernel functions that borrow supports uniformly from the whole sample space without considering the adjacency between samples [10–13], in general, it is very difficult to use kernel functions for factor variables with non-orderable categorical values.

Bayesian estimations are an alternative approach for the probability estimation. For example, the probability distribution of a factor variable is often assumed to have a Dirichlet prior, and estimated as a posterior distribution with pseudo counts [14]. Some previous studies used hierarchical Dirichlet models to address such a problem [15, 16]. However, these studies are limited with only a few categories for each variable, and they also have the limitation for handling the marginal sparsity of sample space. On the other hand, Wong and Ma proposed a nonparametric Bayesian estimation for multivariate data using an optional Pólya tree (OPT) [17]. By adopting optional partitioning and stopping to a Pólya tree, which was originally proposed by Ferguson [18] and investigated further by Lavine [19, 20], an OPT constructs a prior distribution that can be applied to various joint probability distributions. The posterior distribution also forms an OPT that recursively partitions the sample space into subregions where samples are considered to be distributed uniformly. OPTs also have been utilized for the distribution comparison between samples observed from two different conditions [21] and the probability density estimation of multivariate censored data [22]. Additionally, its computational aspects have been investigated to improve the high demands of computing powers [23]. Since approaches using OPTs consider samples in a subregion together to estimate the probability density or mass, they can partially compensate the sparseness of samples and provide robust estimations [17, 21–23].

While the Bayesian estimation with OPT priors provides a good theoretical framework for the joint probability distributions of both continuous and discrete variables, its calculation is not straightforward for factor variables with many categories. To partition the sample space into subregions with uniform distributions, the OPT calculation investigates the possible partitioning options, and assigns a partitioning probability to each option according to the likelihood that the partitioned subregions have uniform distributions. When a region is partitioned into two subregions, the number of possible partitions exponentially increases with the number of categorical values of factor variables. It significantly limits the OPT calculation for factor variables with many categorical values.

In this paper, we propose a Bayesian estimation with OPT priors for the joint probability distribution of multivariate factor variables with many categories, for which kernel approaches cannot be directly applied. The proposed method shrinks the searching space for partitioning options by suggesting suboptimal options based on local marginal populations, and makes the OPT calculation feasible for factors with many categories. In addition, the method enables to estimate probabilities of each combination of categorical values by extending the original OPT to combination cells. The improvement of the method was demonstrated through intensive simulations. Case studies with diagnosis data in intensive care units and genomic data also showed the usefulness of the method in terms of estimating the probability distribution as well as discovering interesting medical hypotheses.

## Methods

### Construction of an OPT with factor variables with many categories

An OPT is the distribution of probability distributions over a multidimensional sample space, defined by a set of probabilities to determine the partitioning of sample spaces and the resulted subregions [17]. To construct an OPT with given data, first, the sample space is recursively partitioned into subregions until the partitioning is meaningless. Then, from the terminal regions by aggregating likelihoods for the sample distributions of subregions, we calculate the probabilities to stop partitioning and for the way of partitioning if partitioned. If samples in a given region are likely to be uniformly distributed, the region has a high probability to stop partitioning. Similarly, among the partitioning choices of a given region, higher probabilities are assigned to ones that make samples in the partitioned subregions more uniformly distributed. In this way, an OPT superposes many trees with different probabilities. A randomly picked tree from an OPT consists of leaf regions that likely have uniform distributions.

For the formal description, consider a sample space of *p* factor variables where $X^{i}\in \left\{x_{1}^{i},x_{2}^{i},\cdots ,x_{n_{i}}^{i}\right\}$ for *i* = 1,2,…,*p*, and a region *A* in the whole sample space. $x_{j}^{i}$ is a categorical value that *X*^*i*^ can have. When factor variable $X^{i}\in \left\{x_{A_{i}(1)}^{i},x_{A_{i}(2)}^{i},\cdots ,x_{A_{i}\left(m_{i}\right)}^{i}\right\}$ in a region *A*, *A* is defined over $\prod_{i=1}^{p}m_{i}$ combinations of categorical values. Here, we denote $A=\prod_{i=1}^{p}\left\{x_{A_{i}(1)}^{i},x_{A_{i}(2)}^{i},\cdots ,x_{A_{i}\left(m_{i}\right)}^{i}\right\}$. To construct an OPT, we apply binary partitioning that divides a region into two subregions, which has been commonly used in previous OPT approaches [17, 21–23]. Assuming that the division occurs for *X*^*i*^, region *A* can be partitioned in $2^{m_{i}-1}$ different ways. In total, $\sum_{i=1}^{p}2^{m_{i}-1}$ partitioning options need be considered. When *m*_*i*,_ the number of categorical values that *X*^*i*^ can have in *A*, is large, it is not possible to investigate the all potential partitions.

To construct an OPT for factors with many categories, the proposed method shrinks the set of partitioning options using marginal populations. If the partitioned subregions have uniform distributions, the marginal distributions of each subregion should be also uniform. While the inverse statement is not always guaranteed, maximizing the marginal uniformity provides a better chance to find subregions with uniform distributions. The detail procedures of the proposed method are as follows.

### (1) Finding suboptimal partitions

Let *N*(*A*) be the number of observed samples in region $A=\prod_{i=1}^{p}\left\{x_{A_{i}(1)}^{i},x_{A_{i}(2)}^{i},\cdots ,x_{A_{i}\left(m_{i}\right)}^{i}\right\}$. To find the suboptimal partition for $X^{i}\in \left\{x_{A_{i}(1)}^{i},x_{A_{i}(2)}^{i},\cdots ,x_{A_{i}\left(m_{i}\right)}^{i}\right\}$, the marginal populations of *X*^*i*^ within *A* is calculated. Let $M_{A}\left(x_{A_{i}(j)}^{i}\right)$ be the marginal population of $x_{A_{i}(j)}^{i}$ of *X*^*i*^, which is given as $M_{A}\left(x_{A_{i}(j)}^{i}\right)=N\left(x_{A_{i}(j)}^{i}\times \prod_{k\neq i}^{}\left\{x_{A_{k}(1)}^{k},x_{A_{k}(2)}^{k},\cdots ,x_{A_{k}\left(m_{k}\right)}^{k}\right\}\right)$. Without loss of generality, we can sort the categorical values so that $M_{A}\left(x_{\left\{A_{i}(1)\right\}}^{i}\right)\leq M_{A}\left(x_{\left\{A_{i}(2)\right\}}^{i}\right)\leq \cdots \leq M_{A}\left(x_{\left\{A_{i}\left(m_{i}\right)\right\}}^{i}\right)$, according to the marginal populations. Here, $x_{\left\{A_{i}\left(j\right)\right\}}^{i}$ denotes the *j*-th ranked categorical value of *X*^*i*^ in *A*. The proposed method partitions *A* over the sorted categorical values at *s*-th ranked categorical value, into $A_{i}^{1}(s)=\left\{x_{\left\{A_{i}\left(1\right)\right\}}^{i},\ldots ,x_{\left\{A_{i}\left(s\right)\right\}}^{i}\right\}\times \prod_{k\neq i}^{}\left\{x_{A_{k}(1)}^{k},x_{A_{k}(2)}^{k},\cdots ,x_{A_{k}\left(m_{k}\right)}^{k}\right\}$ and $A_{i}^{2}(s)=\left\{x_{\left\{A_{i}\left(s+1\right)\right\}}^{i},\ldots ,x_{\left\{A_{i}\left(m_{i}\right)\right\}}^{i}\right\}\times \prod_{k\neq i}^{}\left\{x_{A_{k}(1)}^{k},x_{A_{k}(2)}^{k},\cdots ,x_{A_{k}\left(m_{k}\right)}^{k}\right\}$. In order to measure the uniformity of marginal population in this case, we introduce a metric $T_{X^{i}}(s)$ calculated by

$$T_{X^{i}}(s)=\sum_{j\leq s}{\left(M_{A}\left(x_{A_{i}(j)}^{i}\right)-\frac{\sum_{j\leq s}M_{A}\left(x_{A_{i}(j)}^{i}\right)}{s}\right)}^{2}+\sum_{j>s}{\left(M_{A}\left(x_{A_{i}(j)}^{i}\right)-\frac{\sum_{j>s}M_{A}\left(x_{A_{i}(j)}^{i}\right)}{A(m_{i})-s}\right)}^{2}$$

The metric calculates the sum of marginal population variations within each subregion. The method finds the suboptimal splitting point $s_{i}^{*}$ that minimizes $T_{X^{i}}(s)$. Consequently, *A* is partitioned into $A_{i}^{1}(s_{i}^{*})$ and $A_{i}^{2}(s_{i}^{*})$ if the division occurs for *X*^*i*^. With such a suboptimal partitioning for each factor variable, the proposed OPT calculation only needs to investigate *p* partitioning options instead of $\sum_{i=1}^{p}2^{m_{i}-1}$ options.

### (2) Calculating subregion likelihoods

In a similar way described in previous OPT works [17, 21–23], the likelihood of sample distribution in a region is calculated recursively with those of the subregions. The likelihood of region *A*, Φ(*A*), is given by

$$\Phi \left(A\right)=\rho \Phi_{0}\left(A\right)+\frac{1-\rho }{p}\sum_{i=1}^{p}\frac{B\left(N\left(A_{i}^{1}\left(s_{i}^{*}\right)\right)+\alpha ,N\left(A_{i}^{2}\left(s_{i}^{*}\right)\right)+\alpha \right)}{B\left(\alpha ,\alpha \right)}\Phi \left(A_{i}^{1}\left(s_{i}^{*}\right)\right)\Phi \left(A_{i}^{2}\left(s_{i}^{*}\right)\right)$$

where $A_{i}^{1}\left(s_{i}^{*}\right)$ and $A_{i}^{2}\left(s_{i}^{*}\right)$ are the suboptimal subregions found in the previous step when partitioned for *X*^*i*^. The first term Φ_0_(*A*) is the likelihood that samples are uniformly distributed in *A* when *A* is not partitioned further. It is calculated as $\Phi_{0}\left(A\right)={\mu \left(A\right)}^{-N\left(A\right)}$ where *μ*(*A*) is the Lebesgue measure of region *A*. The second term is the likelihood when *A* is partitioned, which is calculated with weighted likelihoods from binary partitioning for each factor variable. B(·) is a beta function. If two subregions have very different numbers of samples, it is weighted more. *ρ* is the weight between cases with and without further partitioning and *α* is a pseudo count. They are parameters of the OPT prior distribution. While the OPT prior described by Wong and Ma [17] has the larger set of parameters to cover general partitioning strategies and weights among the ways of partitioning, the employed prior distribution in this work has a simplified parameter set to make the OPT calculation feasible.

The whole sample space is partitioned into the suboptimal subregions until the partitioning is meaningless, which is that further partitioning is impossible or subregions have no sample. In such a terminal region, the likelihood is just given as $\Phi \left(A_{terminal}\right)=\Phi_{0}\left(A_{terminal}\right)$. From the terminal regions, the likelihoods of upper-level subregions are subsequently calculated.

### (3) Constructing a posterior OPT distribution

As described in Wong and Ma [17], the posterior distribution is constructed using the likelihoods calculated in the previous step. Briefly, the posterior distribution given data **D** is defined as an OPT with stopping probability to determine whether a given region will be further partitioned or not, selection probabilities for the way of partitioning, and allocated probabilities to each partitioned region. The stopping probability of region *A* is obtained by $\rho \left(A|D\right)=\rho \Phi_{0}\left(A\right)/\Phi \left(A\right)$. The selection probability of partitioning for *X*^*i*^ among the investigated *p* partitioning choices is given proportionally to the likelihood, that is,

$$Pr[SplittingAforX^{i}|D]\propto \frac{B\left(N\left(A_{i}^{1}\left(s_{i}^{*}\right)\right)+\alpha ,N\left(A_{i}^{2}\left(s_{i}^{*}\right)\right)+\alpha \right)}{B\left(\alpha ,\alpha \right)}\Phi \left(A_{i}^{1}\left(s_{i}^{*}\right)\right)\Phi \left(A_{i}^{2}\left(s_{i}^{*}\right)\right)$$

Finally, the probability mass *θ*_1_ and *θ*_2_, which are respectively allocated to the partitioned region $A_{i}^{1}\left(s_{i}^{*}\right)$ and $A_{i}^{2}\left(s_{i}^{*}\right)$, are drawn from a beta distribution with parameters of $N\left(A_{i}^{1}\left(s_{i}^{*}\right)\right)+\alpha$ and $N\left(A_{i}^{2}\left(s_{i}^{*}\right)\right)+\alpha$. The probabilities of the partitioned regions are obtained as $\mathrm{Pr}\left[A_{i}^{1}\left(s_{i}^{*}\right)|D\right]=\frac{\theta_{1}}{\theta_{1}+\theta_{2}}\mathrm{Pr}\left[A|D\right]$ and $\mathrm{Pr}\left[A_{i}^{2}\left(s_{i}^{*}\right)|D\right]=\frac{\theta_{2}}{\theta_{1}+\theta_{2}}\mathrm{Pr}\left[A|D\right]$. Starting from the whole sample space with probability 1, the probabilities of OPT subregions can be calculated recursively.

### (4) Extending to combinatorial cells using uniform Dirichlet distributions

The OPTs described in Wong and Ma and other works [17, 21–23] commonly consist of stopping probabilities $\rho \left(A|D\right)$, selection probabilities $Pr[SplittingAforX^{i}|D]$, and allocated probabilities $\mathrm{Pr}\left[A_{i}^{l}\left(s_{i}^{*}\right)|D\right]$ as described in the above. In addition, the proposed method extends an OPT to unit cells made by combinations of each categorical value. If a subregion of an OPT is not further partitioned, samples in the region are considered to be uniformly distributed. Therefore, the posterior probabilities assigned to cells in the region are allocated from a uniform Dirichlet distribution.

Formally, for a region $A=\prod_{i=1}^{p}\left\{x_{A_{i}(1)}^{i},x_{A_{i}(2)}^{i},\cdots ,x_{A_{i}\left(m_{i}\right)}^{i}\right\}$ with $C(A)=\prod_{i=1}^{p}m_{i}$ cells and *N*(*A*) samples, a vector of probability masses $\left[\theta_{1},\theta_{2},\ldots ,\theta_{C(A)}\right]$ for cells is drawn from a Dirichlet distribution with a parameter set $\left[\frac{N\left(A\right)}{C\left(A\right)}+\beta ,\ldots ,\frac{N\left(A\right)}{C\left(A\right)}+\beta \right]$. Then, the posterior probability of the *j*-th cell $\prod_{i=1}^{p}\left\{x_{A_{i}\left(k_{i}\right)}^{i}\right\}$ in the region is calculated by $\mathrm{Pr}\left[\prod_{i=1}^{p}\left\{x_{A_{i}\left(k_{i}\right)}^{i}\right\}|D\right]=\frac{\theta_{j}}{\sum_{c=1}^{C(A)}\theta_{c}}\mathrm{Pr}\left[A|D\right]$. Here, *β* is another OPT prior parameter for factor variables.

### Inference of the probability distribution from an OPT

Given a posterior OPT distribution over a whole sample space, a tree with fixed terminal partitions and probabilities of combinatorial cells can be randomly chosen according to the stopping probabilities, selection probabilities, and the assigned probabilities of regions and cells. Since each random tree has its own probability to be chosen, the expected probability of any combination of categorical values can be properly calculated. Alternatively, the probability distribution can be estimated from the mode of the posterior OPT distribution as suggested by Wong and Ma [17].

### Approximating OPT calculations

It is well-known that constructing an OPT requires heavy computational resources for its recursive partitioning and likelihood calculations [23]. It is mainly because the exact calculation requires partitioning a sample space to the end until further partitioning is not possible or meaningless. While the proposed method provides the feasible computations for factor variables with many categorical values, the exact calculation of the proposed method still requires a long computation time and a large memory size because of the complex nature of OPT calculations as well as the additional tasks to determine the suboptimal partitioning options and the extension to combination cells.

To improve the computational efficiency of the proposed method, we applied approximated calculations with limited-lookahead OPT (LL-OPT) [23] for the numerical studies of this work. Briefly, the LL-OPT calculates Φ(*A*) with *h*-level further partitioning instead of partitioning to the end, and chooses a tree with the maximum posterior probability among ones growing *q*-level further (*q* ≤ *h*). The LL-OPT calculation is recursively applied to each leaf region within the chosen tree. Such a process is repeated until further partitioning is impossible or meaningless. While *q* is often fixed to be 1 [23], *h* is a tuning parameter for the trade-off between the precision and computation time.

### Availability of software and simulation data

The software package of the proposed method and simulation data used in this paper are freely available at http://cdal.korea.ac.kr/DEFMC. We provide source codes of the implementation as well. For the case studies, we used two public data sets, which are MIMIC-II Clinical Database and TCGA data sets [24, 25]. The MIMIC-II Clinical Database is available at https://physionet.org/physiobank/database/mimic2cdb-ps/, and TCGA data sets are available at https://portal.gdc.cancer.gov/.

## Results and discussion

### Simulation study

We evaluated the proposed method through simulation studies with random samples generated from pre-assumed joint probability density distributions. The discrepancy of the estimated distribution from random samples to the true distribution was measured by the root sum square error (RSSE). The RSSEs of the proposed method were compared with those of the conventional combination-wise estimation that estimates the probability of a category combination by simply counting samples falling into the combination. The simulation studies were performed with three-dimensional factor data under nine different joint distributions with various numbers of categories and population sizes. We commonly employed lookahead parameter *h* = 3 to reduce computation times.

**Fig 1:**
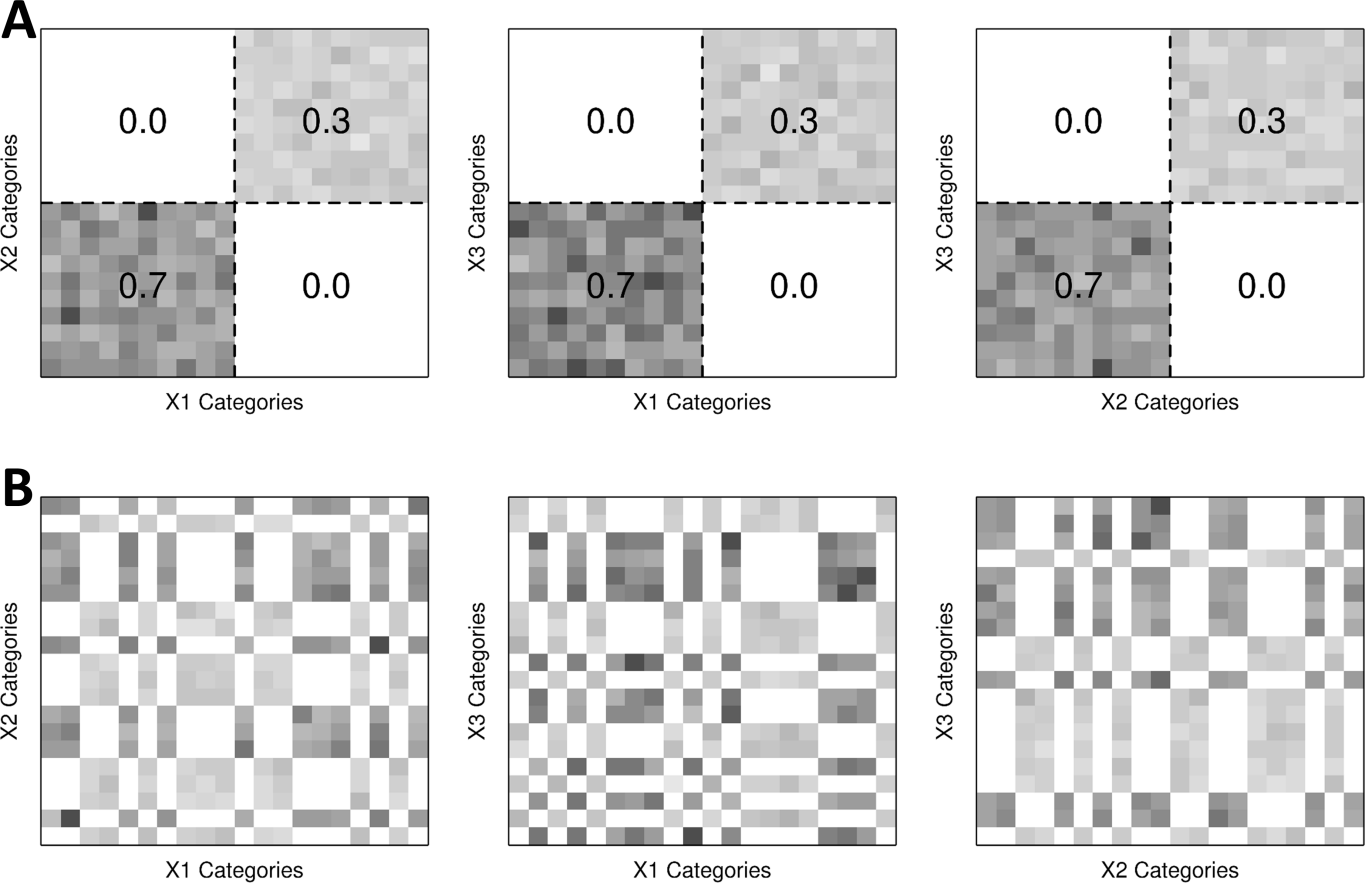
Projections of three-dimensional simulation data of setting (1) to marginal two-dimensional planes. **(A)** Designed data distributions of the three-dimensional data of 20 x 20 x 20 categories from two-level uniform distribution. **(B)** Observed data distributions of the same data by randomly ordered categorical values.

First, the proposed method was evaluated with the joint uniform distributions (Fig 1, Figure B in S1 File, and simulation setting (1) and (2) in S1 File). The sample space is composed of three factor variables $X^{i}\in \left\{x_{1}^{i},x_{2}^{i},\cdots ,x_{m_{i}}^{i}\right\}$ for *i* = 1,2,3. The fine categories of each variable are assumed to be uniformly distributed from two hidden super categories, which means that for $X^{i}\in \left\{X_{\left(1\right)}^{i},X_{\left(2\right)}^{i}\right\}$ with $X_{\left(1\right)}^{i}=\left\{x_{1}^{i},\cdots ,x_{m_{i}/2}^{i}\right\}$ and $X_{\left(2\right)}^{i}=\left\{x_{m_{i}/2+1}^{i},\cdots ,x_{m_{i}}^{i}\right\},\mathrm{Pr}\left[x_{j}^{i}\right]=2p_{i}/m_{i}$ for $x_{j}^{i}\in X_{\left(1\right)}^{i}$ and $\mathrm{Pr}\left[x_{j}^{i}\right]=2(1-p_{i})/m_{i}$ for $x_{j}^{i}\in X_{\left(2\right)}^{i}$, while the association to the super categories are not provided in the data. In the simulation setting (1), *p*_*i*_ = 0.7 for all three variables and the factor variables are perfectly dependent. In other words, the probability of a fine combination $x_{j}^{1}\times x_{k}^{2}\times x_{l}^{3}$ is given as $0.7/\left(\prod_{i}m_{i}/2\right)$ for $x_{j}^{1}\times x_{k}^{2}\times x_{l}^{3}\in \prod_{i}\left\{x_{1}^{i},\ldots ,x_{m_{i}/2}^{i}\right\},0.3/\left(\prod_{i}m_{i}/2\right)$ for $x_{j}^{1}\times x_{k}^{2}\times x_{l}^{3}\in \prod_{i}\left\{x_{m_{i}/2+1}^{i},\ldots ,x_{m_{i}}^{i}\right\}$, and 0 otherwise. Likewise, *p*_1_ = 0.7, *p*_2_ = 0.8, and *p*_3_ = 0.9 in the simulation setting (2), and three factor variables are assumed to be perfectly independent to each other. For example, $Pr\left[x_{j}^{1}\times x_{k}^{2}\times x_{l}^{3}\right]=0.504/\left(\prod_{i}m_{i}/2\right)$ for $x_{j}^{1}\times x_{k}^{2}\times x_{l}^{3}\in \prod_{i}\left\{x_{1}^{i},\ldots ,x_{m_{i}/2}^{i}\right\}$. We generated random samples according to the true distributions, considering the fine categories in a same super category are uniformly distributed. Since the categories of variables are observed without orders in general, the association to the super categories is not observed as shown in Fig 1B We examined the performance of the proposed method for various numbers of fine categories (*m*_*i*_ = 20, 40, 60, 80 and 100 for all three variables) and samples sizes (10,000, 20,000, 30,000, 50,000 and 100,000). We measured the errors of the estimated probabilities to the true probabilities from the assumed distribution for combination cells, and calculated a RSSE. The simulations were repeated 100 times for each simulation setting, and the averages and variances of RSSEs were calculated.

**Fig 2:**
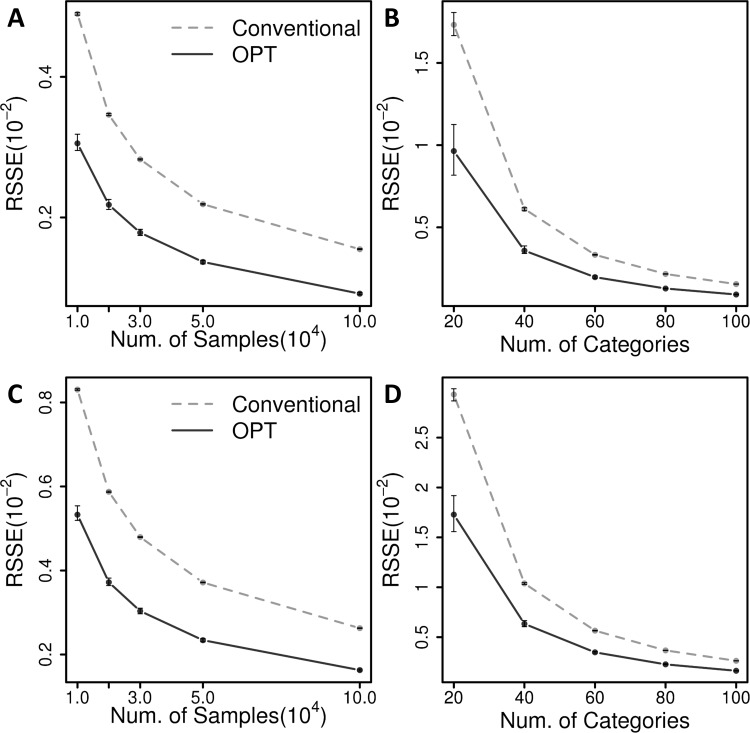
Simulation results with two-level uniform distributions. For simulation setting (1), shown are the RSSEs of the estimated joint probabilities to the true values **(A)** as a function of sample sizes with a fixed number of categories, and **(B)** as a function of various numbers of categories with a fixed sample size. For simulation setting (2), shown are the estimation RSSEs **(C)** as a function of sample sizes and **(D)** as a function of numbers of categorical values. The RSSEs of the proposed method are shown in solid lines, and those of conventional combination-wise estimation are shown in dashed lines. The average RSSEs from 100 repeated simulations are shown with dots and the standard deviation is shown with error bars.

In the simulation setting (1) and (2), the proposed method shows significant improvements for the estimation of joint probabilities compared with naïve combination-wise estimations (Fig 2). When estimated with 10,000 samples for 100^3^ combination cells (i.e. *m*_*i*_ = 100) in the simulation setting (1), the average RSSE to the true probabilities by the proposed method was 0.003 while that of the combination-wise estimation was 0.005. Similarly, for the same condition in the simulation (2), the proposed method resulted in the average RSSE of 0.006 while the conventional method showed 0.010. Fig 2A and 2C show RSSEs of the proposed method and conventional combination-wise estimation for various sample sizes when the number of categorical values of variables is 100. From the baseline performance of the conventional estimations, the proposed method decreased RSSEs for all tested cases, by 36.9~40.9% in the setting (1) and 36.2~37.1% in the setting (2). Fig 2B and 2D show the results for various numbers of categorical values when the sample size is fixed to 0.1 × *m*_1_ × *m*_2_ × *m*_3_. The proposed method also shows improvements across all conditions by reducing RSSEs by 40.8~44.3% for the case (1) and 36.8~39.1% for the case (2) from the conventional estimations. The variances of the RSSEs are quite small for all simulation cases. For example, estimations by the proposed method for 20^3^ cells with 800 samples have the largest coefficient of variances among the tested settings, which is still less than 0.01. The small variances indicate that the proposed method robustly improves the estimation.

**Fig 3:**
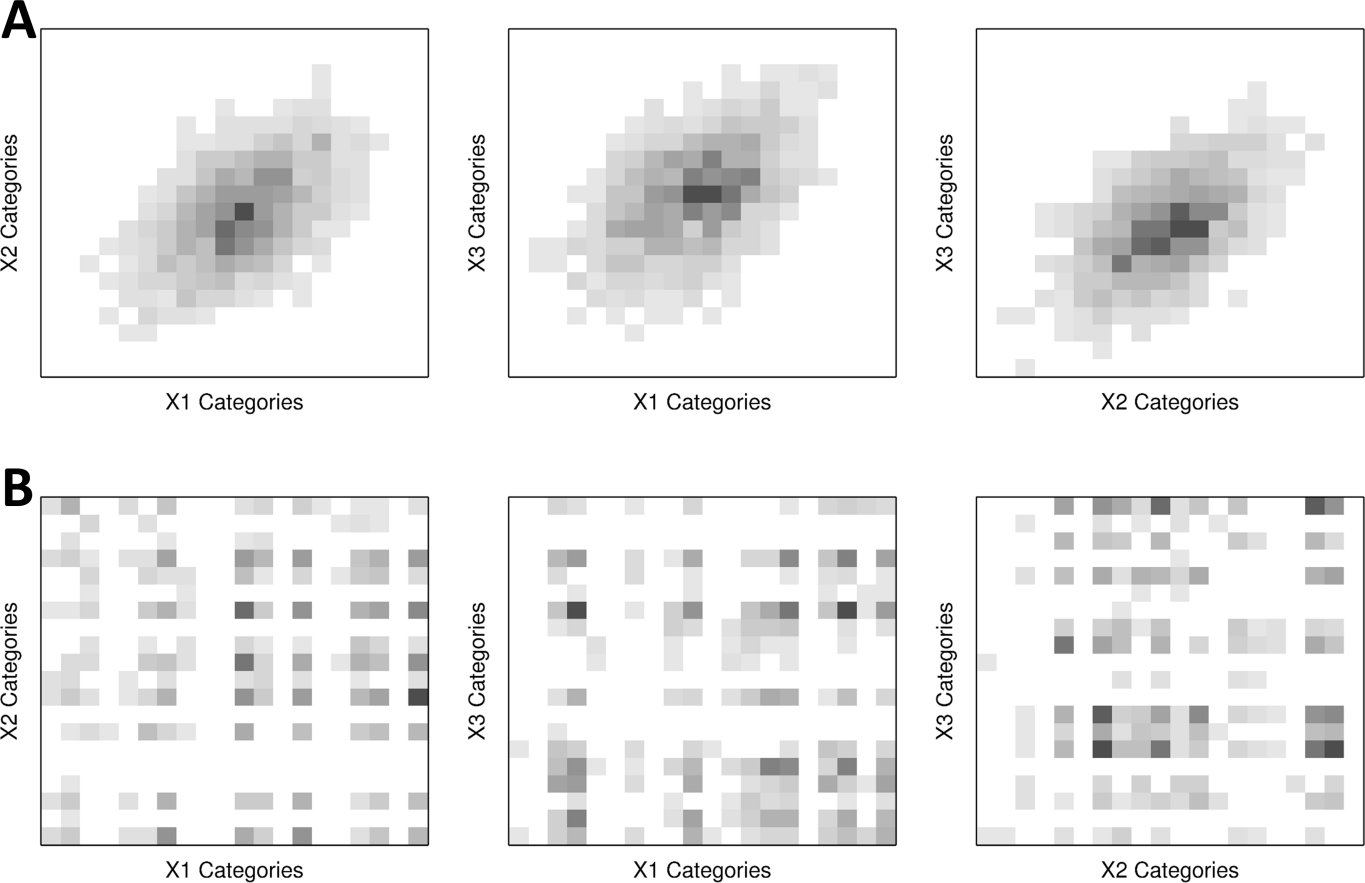
Projections of three-dimensional simulation data of setting (3) to marginal two-dimensional planes. **(A)** Designed data distribution of the three-dimensional data of 20 x 20 x 20 categories from normal distributions. **(B)** Observed data distribution of the same data by randomly ordered categorical values.

**Fig 4:**
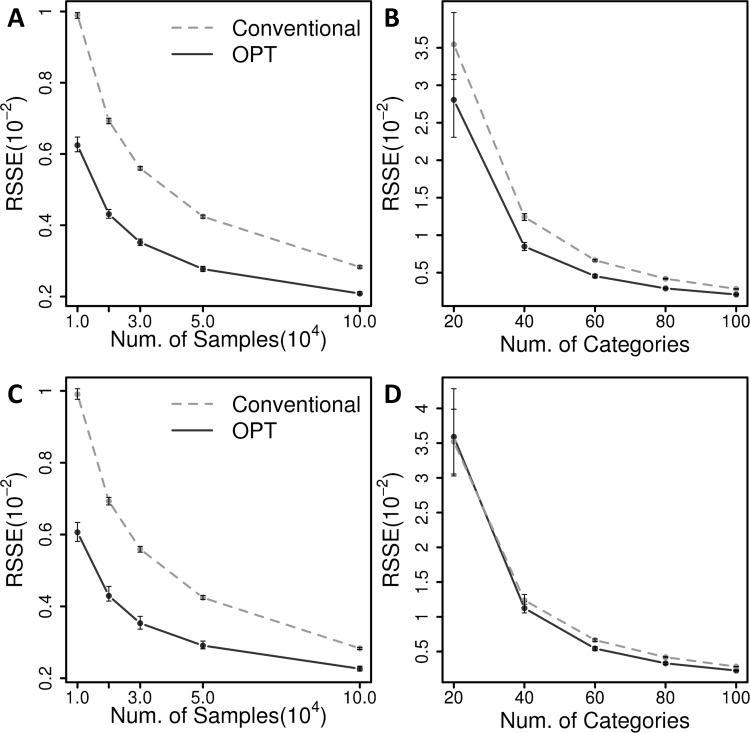
Results of the simulations with normal distributions. For simulation setting (3), shown are the RSSEs of the estimated joint probabilities to the true values **(A)** as a function of sample sizes with a fixed number of categories, and **(B)** as a function of various numbers of categories with a fixed sample size. For simulation setting (4), shown are the estimation RSSEs **(C)** as a function of sample sizes and **(D)** as a function of numbers of categorical values. The RSSEs of the proposed method are shown in solid lines, and those of conventional combination-wise estimation are shown in dashed lines. The average RSSEs from 100 repeated simulations are shown with dots and the standard deviation is shown with error bars.

The proposed method also outperforms the conventional estimation for randomly generated data from joint normal distributions (Figs 3 and 4, Figure C in S1 File, and simulation setting (3) and (4) in S1 File). A trivariate joint normal distribution is discretized over *m*_1_ × *m*_2_ × *m*_3_ uniformly partitioned cells, and random samples are generated accordingly (Fig 3A). The partitioned cells are considered to be combinations of categorical values and random samples are observed without an order (Fig 3B). The method was tested for random data with low and high correlations in the simulation setting (3) and (4), respectively. Similar to the previous cases in simulation setting (1) and (2), the estimations were performed with various numbers of categorical values and sample sizes, and repeated by 100 times for each case. As shown in Fig 4A and 4C, in the simulation with different numbers of samples, the proposed method reduced the error by 26.3~37.8% for the setting (3), and 20.0~38.8% for the setting (4). In the cases with various numbers of categorical values for the simulation setting (3), the RSSEs are reduced by 20.0~31.8% from the baseline of the conventional combination-wise estimations (Fig 4B). In the simulation setting (4), the proposed method results in improved estimation errors by 9.2~21.1% for all cases except one for 20^3^ categorical combinations with 10,000 samples (Fig 4D). Even in this case, the proposed method shows very comparable performance with the conventional estimation. Overall, the most simulation cases show relatively small variances for the estimated errors as like the simulation setting (1) and (2), which also implies the robust improvements by the proposed method.

Furthermore, we examined the performance of our method for various distributions, which are joint normal distributions with no correlation, additive exponential distributions, a combination of Clayton and uniform distributions, log-normal distributions with high and low correlations, and joint uniform distributions with 5, 10 and 20 hidden super categories (simulation setting (5) to (12) in S1 File). In addition to the conventional combination-wise estimation, the proposed method was compared with a kernel density estimation (KDE) for categorical variables, which smooths the probability distribution across the whole sample space [10–12]. In the KDE, the smoothness is controlled by kernel bandwidth. Here, we adapted kernel bandwidth (0.9), according to the prior works with a few categories [13]. To evaluate the proposed partitioning based on marginal populations, we additionally compared with a variation of the proposed method that randomly partitions the sample space. The random partitioning version of the proposed method separates a given region into two subregions by randomly selecting categorical values instead of finding the suboptimal partitioning in the step (1) of the proposed procedure described in Methods section. The following procedures are identical with the proposed method.

In the extended simulation study, the proposed method outperforms other compared methods for the most cases (Table 1). Our method shows the lowest RSSEs for all the simulation cases. The estimation errors are improved by 2.2% ~ 40.8% from the conventional combinational-wise estimations, and by 23.9% ~ 93.6% from KDE methods. As observed in the previous cases, the estimation RSSEs from repeated simulations commonly have small variances relatively to the average improvements, which presents that the proposed method can robustly reduce the estimation error.

**Table 1:** Estimation errors of the simulation study cases.

Simulation Setting	Number of Samples	Error (RSSE × 10^4^)			
		OPT	Conv.	KDE	Rand OPT
**Case (1)**	25,000	**19.54**±**0.22**	30.95±0.04	28.62±0.04	23.00±0.26
	50,000	**13.67**±**0.11**	21.89±0.03	20.46±0.03	16.07±0.22
	100,000	**9.16**±**0.07**	15.48±0.02	14.70±0.02	10.48±0.09
**Case (2)**	25,000	**33.22**±**0.31**	52.53±0.05	47.29±0.05	36.18±0.50
	50,000	**23.45**±**0.21**	37.14±0.04	33.45±0.03	26.12±0.24
	100,000	**16.27**±**0.08**	26.27±0.02	23.67±0.02	18.26±0.17
**Case (3)**	25,000	**38.39**±**0.39**	61.63±0.21	63.64±0.79	41.61±0.33
	50,000	**27.53**±**0.26**	42.43±0.16	46.28±0.80	30.60±0.37
	100,000	**20.76**±**0.16**	28.29±0.10	31.60±0.56	23.94±0.28
**Case (4)**	25,000	**38.49**±**0.62**	61.62±0.35	58.14±0.21	47.64±0.66
	50,000	**29.27**±**0.43**	42.44±0.24	62.98±2.43	36.48±0.44
	100,000	**22.55**±**0.30**	28.30±0.15	36.43±0.98	28.87±0.45
**Case (5)**	25,000	**37.06**±**0.38**	61.65±0.18	59.30±0.36	40.43±0.38
	50,000	**25.92**±**0.20**	42.43±0.15	45.97±0.76	28.52±0.33
	100,000	**19.01**±**0.10**	28.29±0.09	32.59±0.70	21.58±0.23
**Case (6)**	25,000	**46.37**±**1.31**	61.64±0.84	128.74±7.16	48.03±1.19
	50,000	**36.56**±**1.18**	42.38±0.63	144.33±10.18	38.82±0.96
	100,000	**27.12**±**0.68**	28.26±0.37	129.34±9.80	28.06±0.80
**Case (7)**	25,000	**42.58**±**0.71**	61.70±0.59	424.61±36.20	47.70±1.18
	50,000	**33.24**±**0.44**	42.45±0.42	391.50±34.31	34.83±0.85
	100,000	**25.97**±**0.28**	28.27±0.23	429.77±38.70	26.16±0.61
**Case (8)**	25,000	**40.04**±**0.27**	60.91±0.11	60.19±0.52	45.12±0.36
	50,000	**28.60**±**0.15**	42.38±0.07	44.46±0.71	32.40±0.27
	100,000	**21.06**±**0.06**	28.26±0.05	34.13±1.00	23.34±0.13
**Case (9)**	25,000	**39.80**±**0.36**	60.94±0.17	63.32±0.86	45.71±0.44
	50,000	**28.69**±**0.19**	42.35±0.10	49.56±1.21	33.23±0.34
	100,000	**21.45**±**0.10**	28.30±0.07	40.55±1.63	24.31±0.12
**Case (10)**	25,000	**33.85**±**2.46**	53.99±3.94	48.60±3.53	37.53±2.83
	50,000	**23.70**±**1.83**	37.862.74	34.10±2.44	27.04±2.29
	100,000	**16.69**±**1.36**	26.86±2.05	24.20±1.83	19.01±1.81
**Case (11)**	25,000	**32.68**±**1.79**	52.49±2.65	47.32±2.36	36.74±1.98
	50,000	**23.04**±**0.99**	37.01±1.57	33.38±1.37	26.47±1.37
	100,000	**16.50**±**0.96**	26.67±1.39	24.04±1.23	19.06±1.23
**Case (12)**	25,000	**29.87**±**0.55**	50.19±1.97	45.72±1.67	34.86±1.34
	50,000	**22.27**±**0.76**	36.83±1.18	33.34±1.02	26.34±0.76
	100,000	**16.10**±**0.55**	26.62±0.87	24.06±0.75	19.08±0.65

For the simulation setting (1) to (12), shown are the averages and standard deviations of RSSEs by the proposed method (OPT), the conventional combination-wise estimation (Conv), kernel density estimation (KDE), and OPT with random partitioning (Rand OPT).

Since the proposed method sorts the categories by their marginal population at each partitioning, it can construct OPTs more efficiently than the random partitioning process. The tree depth of the proposed method is expected to be lower than the OPT with random partitioning, which means advantages in the computation time and performance.

We evaluated the computation time of the methods for each simulation setting (Table A in S1 File). The experiment result shows that the proposed method is feasible under extreme conditions, such as 100^3^ cells with 100,000 samples, since the proposed method is computed in under 100 seconds for all simulation settings. As expected, the computation time for proposed method was lower than those of random OPT for all simulation settings; the computation time was reduced by 33.6% in average.

### Case study I

We applied the proposed method to the analysis of real data from MIMIC-II Clinical Database [24]. The database is freely available on the website for MIMIC-II research (https://physionet.org/physiobank/database/mimic2cdb-ps/). The data set includes primary and secondary diagnosis of 4,928 patients in intensive care units. The patient diagnosis is encoded by ICD-9 codes that classify diseases in a hierarchical structure. By employing the second-level diagnosis codes, each of the primary and secondary diagnosis is presented by a factor variable with 94 categorical values. In total, the data set is presented by 94^2^ combination cells.

To demonstrate the effectiveness of the proposed method, we estimated the joint probabilities from 500 samples randomly chosen from the whole data set. The objective of this experiment is to evaluate whether the method can reconstruct the original sample distribution given the restricted number of samples. Since the whole 4,928 observations is the largest data set we have, we tested whether the proposed method can estimate the sample distribution of the whole data set from randomly subsampled data. In other words, the probabilities estimated with the whole data set are considered to be true probabilities, and randomly chosen data are the samples for the density estimation experiments. We compared the proposed method with the conventional combination-wise estimation.

We repeated this implementation 100 times and calculated RSSEs of the estimated distributions. Since the random sample density of this experiment (500/94^2^) is very similar to those of the simulation cases with 50,000 samples for 100^3^ combinations in the previous section, we employed the same lookahead parameter, *h* = 3.

**Fig 5:**
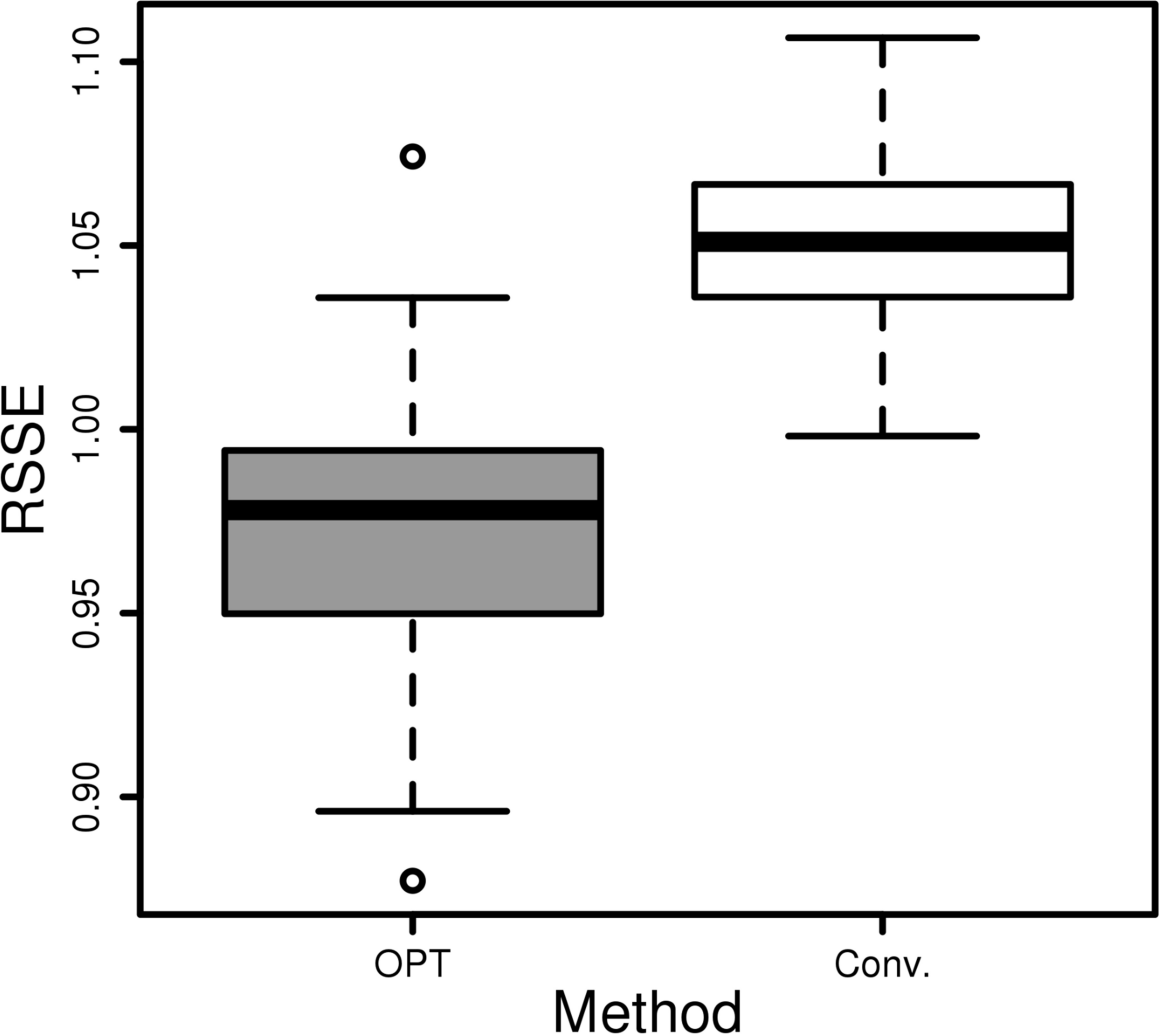
Probability estimation comparison for the diagnosis data of intensive care unit patients. Estimation RSSEs to the true probabilities by the proposed method (OPT) and the conventional combination-wise method (Conv). Estimations were repeated by 100 times with randomly selected subsets of samples.

In the analysis of the diagnosis data, the proposed method has a lower estimation error than the conventional calculation (Fig 5). Compared with the conventional combination-wise estimation, the proposed method reduced the average RSSE by 7.5%, which is a statistically significant improvement (p-value < 10^−12^). Importantly, the partitioning pattern by the proposed method can provide useful intuitions for the analysis of biomedical data. The proposed method partitions the whole sample space into subregions within which combinations of categorical values are uniformly distributed. The conditional uniformness of categories implies that the categorical values in the same terminal region might have certain relations that are observed only with the given condition. For example, in the partitioning pattern of the diagnosis data, CEREBROVASCULAR DISEASE and OTHER DISEASES OF RESPIRATORY SYSTEM are commonly bound as secondary diagnosis when CEREBROVASCULAR DISEASE, COMPLICATIONS OF SURGICAL AND MEDICAL CARE, NOT ELSEWHERE CLASSIFIED, and OTHER FORMS OF HEART DISEASE are given as primary diagnosis. It might indicate that potential relatedness between CEREBROVASCULAR DISEASE and OTHER DISEASES OF RESPIRATORY SYSTEM that occurs only in such conditions while the two diseases are totally separated in the disease classification of ICD-9.

**Fig 6:**
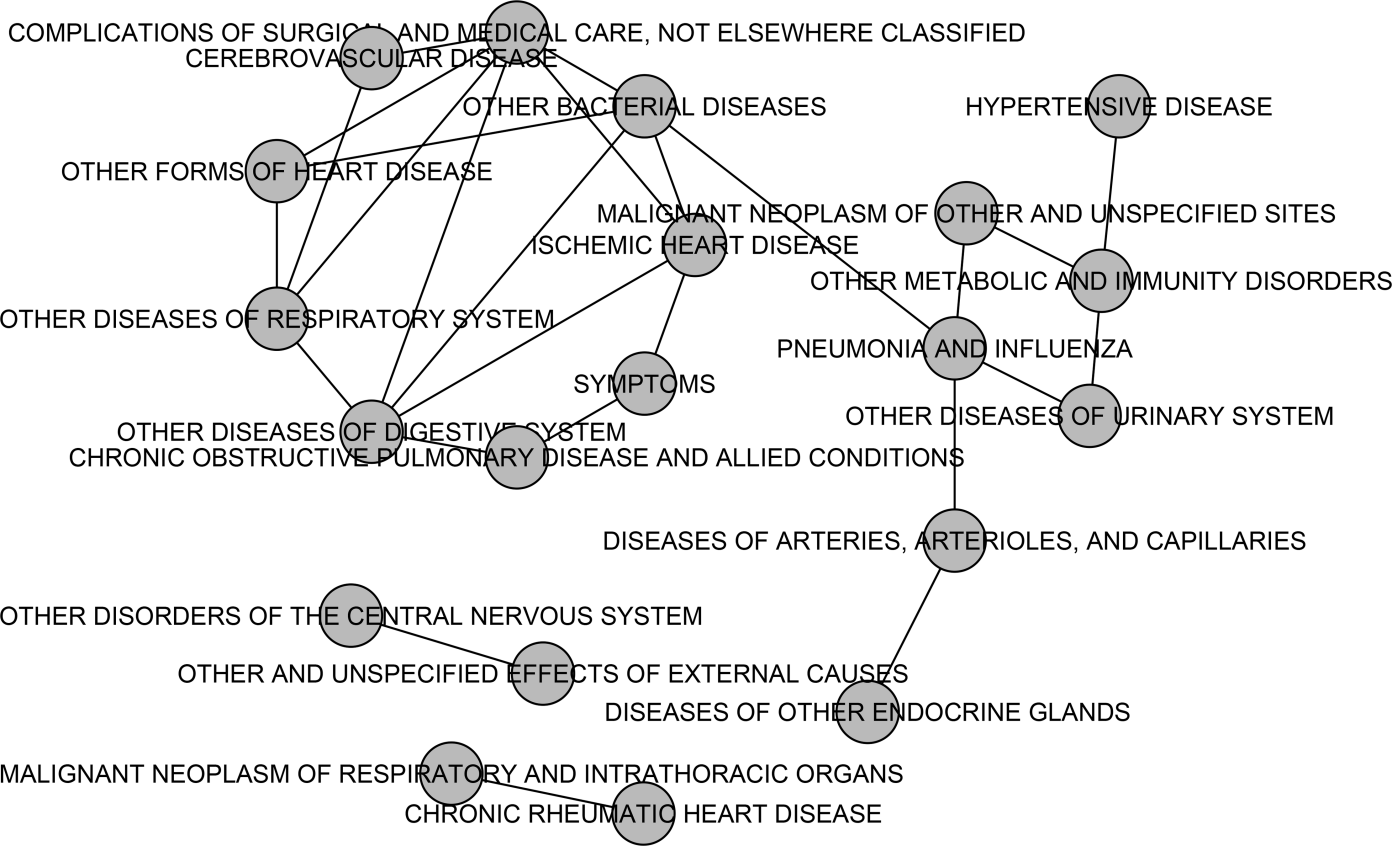
A disease network constructed from partitioning patterns of the proposed method. The top 20 most frequently coexisting categorical values in the same terminal regions of the partitioning tree constructed by the proposed method.

From the analysis of the diagnosis data with the proposed method, a disease network can be constructed by linking disease codes that are frequently bound together in the same terminal regions (Fig 6). The coexistence in the terminal regions with zero sample is not considered. Fig 6 shows top 20 most frequently coexisting relations in the diagnosis data. In this network, COMPLICATIONS OF SURGICAL AND MEDICAL CARE, ISCHEMIC HEART DISEASE, OTHER BACTERIAL DISEASES, and OTHER DISEASES OF THE DIGESTIVE SYSTEM are shown to be related to each other. Some relations found in the network are partially supported by previous studies. For example, relationship between heart and respiratory diseases is observed from an edge between OTHER FORMS OF HEART DISEASE and OTHER DISEASES OF RESPIRATORY SYSTEM. This detection accord with some recent studies that investigate the relation between heart and respiratory diseases. Apostolo *et al*. demonstrates that lung function abnormalities are a common symptom for chronic heart failure [26]. Van Eeden *et al*. argues that lung inflammation is an important factor for heart diseases [27]. The overall analysis implies that the proposed method has a potential to provide interesting medical hypothesis that can be further investigated.

### Case study II

We applied the proposed method to estimate the probability distribution of RNA-Seq read counts, which can be used for gene expression calculation. Since a sequencing read count matrix consist of observations from gene-sample combinations, it can be considered as a contingency table with two categorical variables. We evaluated the proposed method on the read count matrix to reconstruct the whole sample space with restricted observations, which is a similar setting to the experiments in the previous section.

In this study, TCGA-BRCA data set is used [25]. The read count matrix of TCGA-BRCA is composed of 20,502 genes and 878 samples for breast cancer. The number of total sequencing reads is 82.9 billion. Consequently, the whole sample space is a 20,502 × 878 matrix with 82.9 billion observations.

**Fig 7:**
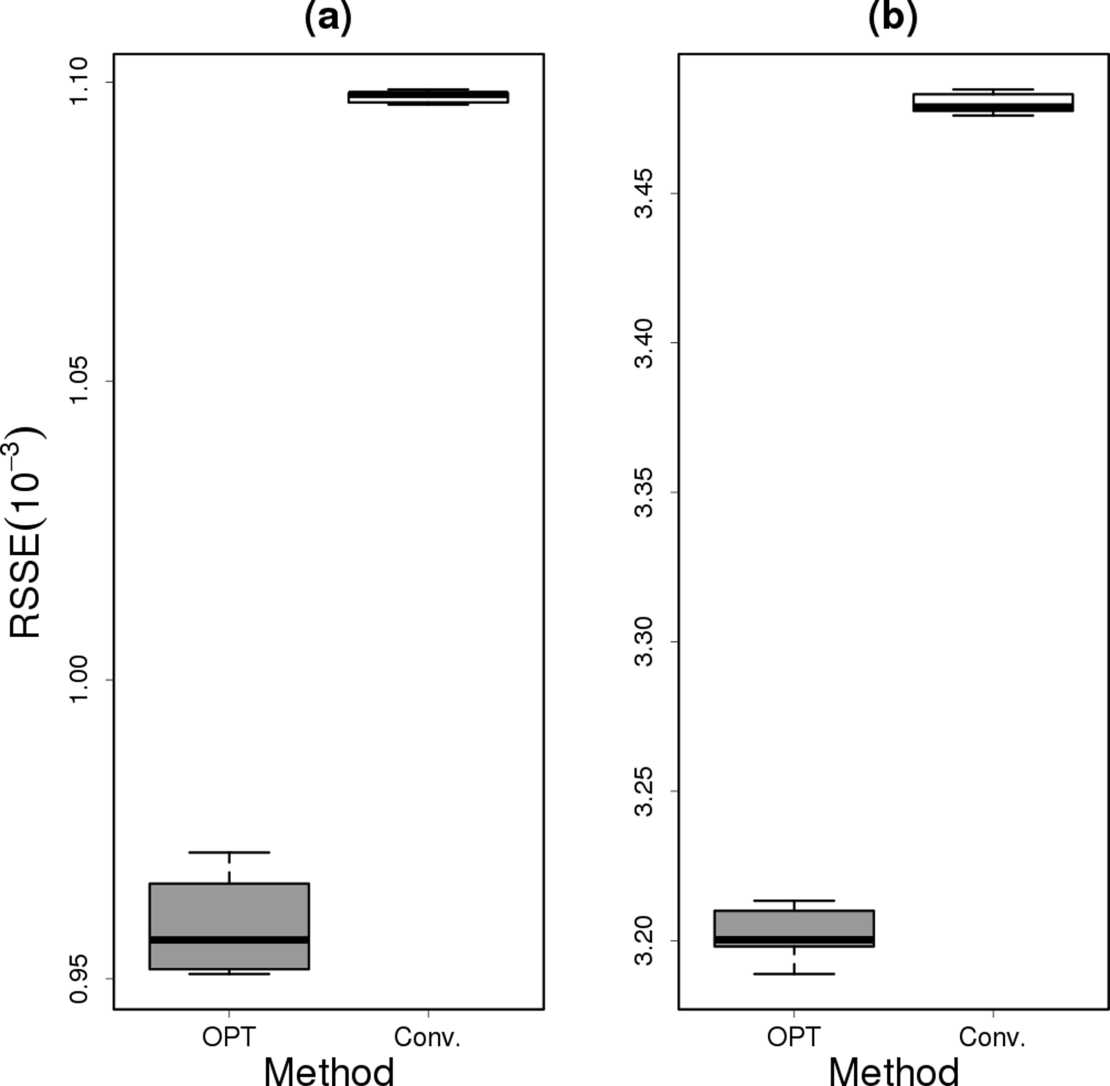
Probability estimation comparison for the read count matrix. The experiment is conducted under the two settings using **(a)** $10^{-5}$ and **(b) $10^{-6}$** of the data set. OPT denotes the proposed method.

The objective of this experiment is to estimate the counting density of sequencing reads. The density distribution obtained from the whole 82.9 billion reads is considered as the true distribution. The proposed method is applied to estimate the true distribution from a random subset of the whole data set. The estimation error is calculated by RSSE as like the previous experiments. The experiment is conducted under two settings, which use 10^−5^ and 10^−6^ of the whole observations. For each setting, we repeated the experiment ten times. As shown in Fig 7, the proposed method significantly outperforms the conventional method. For example, the RSSE is reduced to 9.57 × 10^−4^ in the experiment using 10^−5^ of the observations, which corresponds to 12.7% of improvement to estimate the counting density. This result implies a possibility to estimate the gene expression indices from an improved counting read density with fewer sequencing reads.

## Conclusion

In this work, we propose an estimation method for the joint probability distribution among multiple factor variables that can have many categorical values. We demonstrate the effectiveness of the proposed method through simulation and case studies. Our method significantly reduces the estimation errors for the all simulation cases. The robustness of the estimation is implied by small variances of estimation errors from a wide range of simulated cases. For a case study with real data, we applied the proposed method to the analysis of diagnosis data of patients in intensive care units. The estimation from subsampling also presented the accuracy and robustness of the proposed method. Moreover, by detecting the conditional uniformness of categorical values, the partitioning pattern by the proposed method has a potential to generate interesting hypotheses for hidden relations among categories, which can be visualized as a network.

We expect that the proposed method can be applied for analyzing a large matrix of observed counts with a little modification because a matrix can be considered as a sample space between two factor variables. Count matrices can be from counts of DNA molecules for thousands of genes from hundreds of patients in high-throughput sequencing [28–30], personalized purchases for millions of items of thousands of users in business [31, 32] and many other areas. The proposed method will be useful for smoothing, decomposing, and factorizing such a matrix. We will extend this work to develop efficient algorithms for matrix analyses.

## Supporting information

S1 File Supplementary information. Detailed simulation settings are described. The Table A, Figs B and C are provided.
(PDF)
