# Supplementary material for: The estimation of probability distribution for factor variables with many categorical values
Source: PLoS One. 2018 Aug 24;13(8):e0202547. doi: 10.1371/journal.pone.0202547 (PMC6108477; doi:10.1371/journal.pone.0202547)
Supplement: S1 File — Detailed simulation settings are described. The Table A, Figs B and C are provided. (PDF) [file pone.0202547.s001.pdf]

Supplementary Information

The Estimation of Probability Distribution for Factor Variables with Many Categorical Values

Minhyeok Lee[1], Yeong Seon Kang[2], Junhee Seok[1*]

[1] School of Electrical Engineering, Korea University, 145 Anam-ro, Seongbuk-gu, Seoul, 02841, South Korea

[2] Department of Business Administration, University of Seoul, 163 Seoulsiripdaero, Dongdaemun-gu, Seoul, 02504, South Korea

[*] Corresponding author: Junhee Seok, jseok14@korea.ac.kr

Supplementary Methods

Simulation Settings

For all these simulation settings, the sample spaces are composed of three factor variables $X^i \in \{x_1^i, x_2^i, \cdots, x_{m_i}^i\}$ for $i = 1,2,3$, where m_i denotes the number of categories of X^i.

1. Joint Uniform Distributions

Each variable is assumed to have two hidden super categories, which means $X^i \in \{X_{(1)}^i, X_{(2)}^i\}$ where $X_{(1)}^i = \{x_1^i, \cdots, x_{m_i/2}^i\}$ and $X_{(2)}^i = \{x_{m_i/2+1}^i, \cdots, x_{m_i}^i\}$. And the probabilities of each fine category that is in a same super category are assumed to be equal. That is, $\Pr[x_j^i] = p_i/(0.5 \times m_i)$ for $x_j^i \in X_{(1)}^i$ and $\Pr[x_j^i] = (1-p_i)/(0.5 \times m_i)$ for $x_j^i \in X_{(2)}^i$.

Simulation setting (1): Perfectly dependent uniform distribution. Three variables are perfectly dependent and $p_i = 0.7$ for all i. That is, $\Pr[x_j^1 \times x_k^2 \times x_l^3] = 0.7/\mathrm{N}_c(\prod_i X_{(1)}^i)$ for the combinations of fine categories $x_j^1 \times x_k^2 \times x_l^3 \in \prod_i X_{(1)}^i$, $\Pr[x_j^1 \times x_k^2 \times x_l^3] = 0.3/\mathrm{N}_c(\prod_i X_{(1)}^i)$ for $x_j^1 \times x_k^2 \times x_l^3 \in \prod_i X_{(2)}^i$, and 0 otherwise, where $\mathrm{N}_c(\cdot)$ denotes the number of unique combination of fine categories that a certain space can compose.

Simulation setting (2): Independent uniform distribution. Three variables are independent, and $p_1 = 0.9$, $p_2 = 0.8$ and $p_3 = 0.7$. That is, for examples, for the combinations of fine categories $x_j^1 \times x_k^2 \times x_l^3 \in \prod_i X_{(2)}^i$, $\Pr[x_j^1 \times x_k^2 \times x_l^3] = 0.006/\mathrm{N}_c(\prod_i X_{(1)}^i)$.

Simulation setting (10), (11) and (12): Independent uniform distribution with multiple super categories. Three variables are independent, and each variable has 5, 10 and 20 super categories for simulation setting (10), (11) and (12), respectively. The probabilities for super categories are randomly assigned for each implementation: $\Pr(X_{(a)}^i) = Unif(0,1)/\sum_a \Pr(X_{(a)}^i)$, where *a* denotes an index for a super category.

2. Normal Distributions

In this simulation, a trivariate joint normal distribution is assumed. In order to generate the simulation data, we assume that the marginal populations of the simulation data follow normal

distributions with the hidden orders of the categories. We discretize the continuous normal distribution, and the probability that is assigned to each combinatorial cell is assumed to be the hidden true probability of the normal distribution with factor variables.

Simulation setting (3): Normal distribution with low correlation. The random sample data are generated from the distribution of $\begin{pmatrix} X^1 \\ X^2 \\ X^3 \end{pmatrix} \sim \mathrm{N}\left\{\begin{pmatrix} 0 \\ 0 \\ 0 \end{pmatrix}, \begin{pmatrix} 1 & 0.49 & 0.49 \\ 0.49 & 1 & 0.49 \\ 0.49 & 0.49 & 1 \end{pmatrix}\right\}$.

Simulation setting (4): Normal distribution with high correlation. The random sample data are generated from the distribution of $\begin{pmatrix} X^1 \\ X^2 \\ X^3 \end{pmatrix} \sim \mathrm{N}\left\{\begin{pmatrix} 0 \\ 0 \\ 0 \end{pmatrix}, \begin{pmatrix} 1 & 0.81 & 0.81 \\ 0.81 & 1 & 0.81 \\ 0.81 & 0.81 & 1 \end{pmatrix}\right\}$.

Simulation setting (5): Independent normal distribution. The random sample data are generated from the distribution of $X^1 \sim \mathrm{N}(0,1)$, $X^2 \sim \mathrm{N}(0,1)$ and $X^3 \sim \mathrm{N}(0,1)$.

3. Other Distributions

Simulation setting (6): Additive exponential distribution. Each variable follows the distribution respectively, $X^1 \sim \mathrm{Exp}(1)$, $Z^1 \sim \mathrm{Exp}(1)$, $Z^2 \sim \mathrm{Exp}(1)$, $X^2 \sim X^1 + Z^1$ and $X^3 \sim X^2 + Z^2$.

Simulation setting (7): Clayton model with uniform distribution. The distribution with two factor variables (X^2, X^3) follows the distribution where survival function is: $S(x^2, x^3) = \left(\mathrm{Exp}\left\{\frac{x^2}{\theta}\right\} + \mathrm{Exp}\left\{\frac{x^3}{\theta}\right\}\right)^{-\theta}$, where $\theta = 0.5$. And the other factor variable follows the distribution of $X^1 \sim 3 \times \mathrm{U}\{x_1^1, x_{m_i}^1\} + 0.1X^2 + 0.1X^3$.

Simulation setting (8): Lognormal distribution with low correlation.

Continuous normal distribution is discretized for the simulation with the lognormal distribution same as the simulations with normal distributions. And the distribution of the sample space follows as $\log\begin{pmatrix} X^1 \\ X^2 \\ X^3 \end{pmatrix} \sim \mathrm{N}\left\{\begin{pmatrix} 0 \\ 0 \\ 0 \end{pmatrix}, \begin{pmatrix} 1 & 0.5 & 0.5 \\ 0.5 & 1 & 0.5 \\ 0.5 & 0.5 & 1 \end{pmatrix}\right\}$.

Simulation setting (9): Lognormal distribution with high correlation. The distribution of the

sample space follows as $\log\begin{pmatrix} X^1 \\ X^2 \\ X^3 \end{pmatrix} \sim \mathrm{N}\left\{\begin{pmatrix} 0 \\ 0 \\ 0 \end{pmatrix}, \begin{pmatrix} 1 & 0.8 & 0.8 \\ 0.8 & 1 & 0.8 \\ 0.8 & 0.8 & 1 \end{pmatrix}\right\}$.

Supplementary Table

Table A. Computation time for the simulation study cases. For the simulation setting (1) to (12), shown are the averages of computation time by the proposed method (OPT), kernel density estimation (KDE), and OPT with random partitioning (Rand OPT). Computation time of the conventional method is zero since we supposed that sample distributions are given for the simulation.

**Simulation Setting**	**Number of Samples**	**Computation Time (seconds)**		
		OPT	KDE	Rand OPT
**Case (1)**	25,000	27.28	0.03	38.03
	50,000	41.56	0.04	61.44
	100,000	62.85	0.03	97.30
**Case (2)**	25,000	36.83	0.04	48.41
	50,000	58.45	0.04	80.12
	100,000	90.61	0.04	134.00
**Case (3)**	25,000	17.39	0.02	24.09
	50,000	26.23	0.02	38.08
	100,000	43.90	0.03	66.72
**Case (4)**	25,000	13.78	0.02	20.50
	50,000	21.34	0.02	32.87
	100,000	36.32	0.02	58.41
**Case (5)**	25,000	17.87	0.02	24.63
	50,000	28.37	0.03	41.28
	100,000	44.77	0.02	67.49
**Case (6)**	25,000	8.42	0.01	14.81
	50,000	13.81	0.01	26.09
	100,000	25.97	0.01	50.23
**Case (7)**	25,000	10.23	0.01	14.98
	50,000	17.09	0.01	27.81
	100,000	31.72	0.01	52.56

**Case (8)**	25,000	33.60	0.03	44.18
	50,000	52.65	0.03	72.63
	100,000	81.99	0.03	123.32
**Case (9)**	25,000	29.47	0.03	41.35
	50,000	45.68	0.03	67.36
	100,000	72.66	0.03	111.74
**Case (10)**	25,000	38.14	0.04	51.20
	50,000	59.73	0.03	87.79
	100,000	93.22	0.03	144.18
**Case (11)**	25,000	37.43	0.04	53.84
	50,000	59.25	0.03	90.33
	100,000	94.23	0.03	158.03
**Case (12)**	25,000	37.30	0.03	53.84
	50,000	59.34	0.03	94.63
	100,000	92.30	0.03	162.82

Supplementary Figures

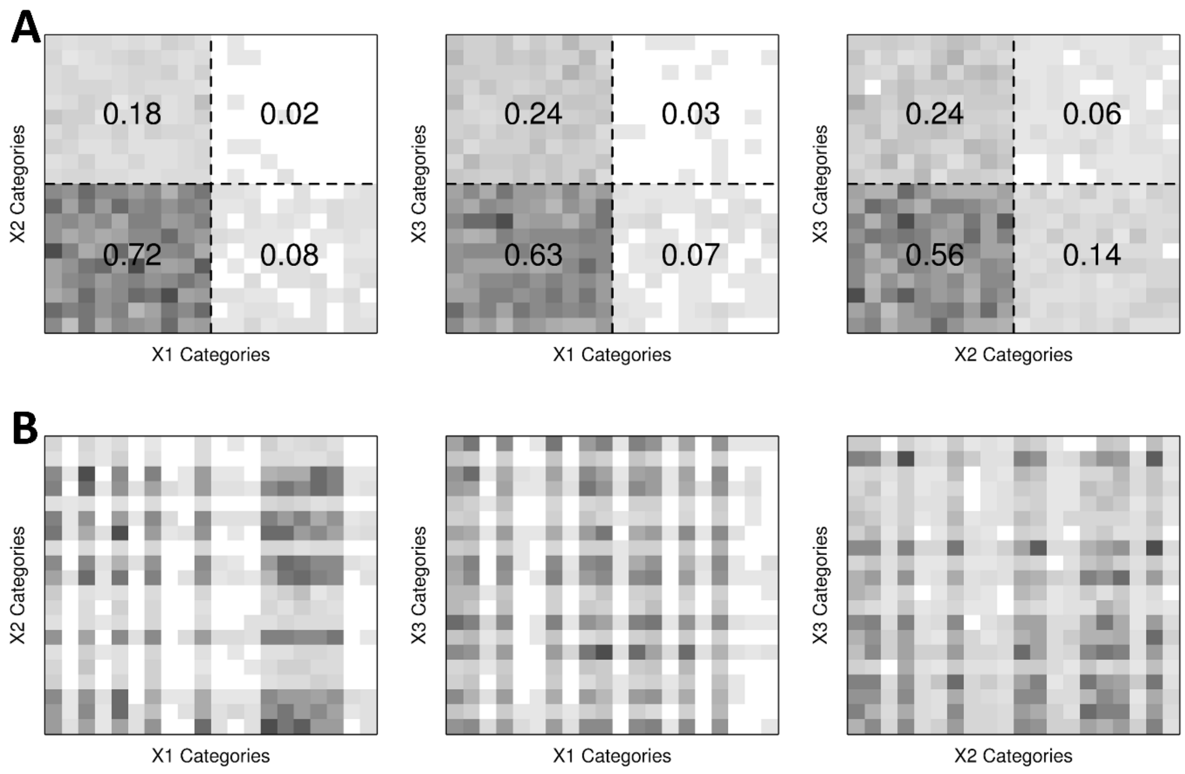

Figure B. Projections of three-dimensional simulation data of setting (2) to marginal two-dimensional planes. (A) Designed data distributions of the three-dimensional data of 20 x 20 x 20 categories from two-level uniform distribution. **(B)** Observed data distributions of the same data by randomly ordered categorical values.

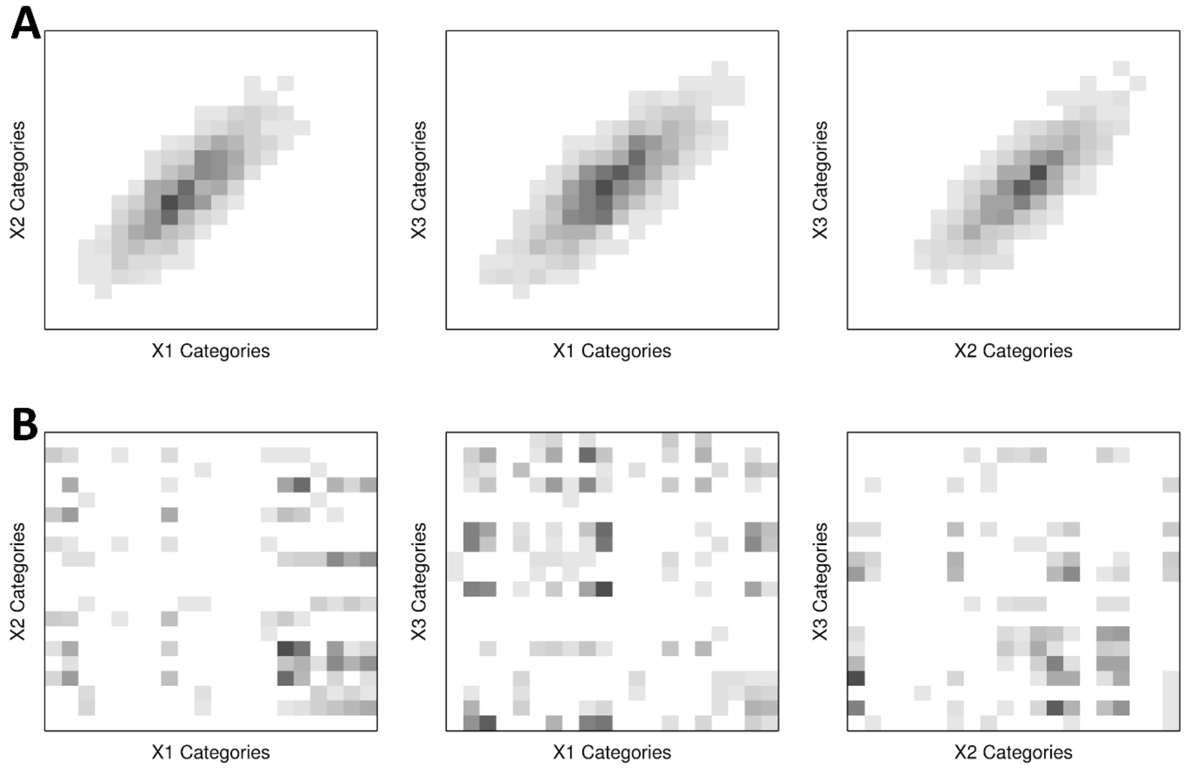

Figure C. Projections of three-dimensional simulation data of setting (4) to marginal two-dimensional planes. (A) Designed data distribution of the three-dimensional data of 20 x 20 x 20 categories from normal distributions. **(B)** Observed data distribution of the same data by randomly ordered categorical values.
